# Study on the Browning Mechanism of Multivitamin Iron Oral Solution Based on Sucrose-Lysine Maillard Reaction

**DOI:** 10.3390/molecules31071087

**Published:** 2026-03-26

**Authors:** Caifeng Su, Jianping Zhu, Zhuangwei Liu, Juying Tan, Jie Jiang, Zhuang Zhao

**Affiliations:** 1 School of Pharmacy, Guangxi Medical University, Nanning 530021, China; caifeng5178@163.com; 2 Guangxi Institute for Drug Control, Nanning 530021, China; zhujianpingorcid@163.com (J.Z.); liuzhuangweill@163.com (Z.L.); tanjuyingtjy@163.com (J.T.)

**Keywords:** Multivitamin Iron Oral Solution, browning mechanism, Maillard reaction, sucrose-lysine, 5-Hydroxymethylfurfural, citric acid, UPLC-Q-TOF-MS/MS

## Abstract

Severe browning often occurs in Multivitamin Iron Oral Solution during storage, which directly leads to the decline of product quality. To clarify the main mechanism of browning in this preparation, the contents of 5-hydroxymethylfurfural (5-HMF) and carbohydrates, as well as the relevant characteristic parameters such as color and fluorescence, were determined at different storage times in this study. Subsequently, four reaction models, namely sucrose-lysine, sucrose-citric acid, sucrose-niacin, and sucrose-folic acid, were constructed according to the formulation of the preparation to systematically investigate the effects of each system on browning. The results showed that the sucrose-lysine model was the main color-forming reaction system of the preparation. Citric acid could significantly promote the hydrolysis of sucrose to produce two reducing sugars, glucose and fructose, which not only provided sufficient substrates for the Maillard reaction (MR), but also led to the massive accumulation of 5-HMF. Further analysis revealed that heating temperature and heating time were significantly positively correlated with the contents of 5-HMF, browning index (BI), color density (CD), and reducing sugars in the solution, while significantly negatively correlated with sucrose content (*p* < 0.05). Two fractions, P1 and P2, were isolated by Sephadex LH-20 column chromatography. Among them, P1 with a molecular weight of 61,660 Da was identified as the key fluorescent color-forming component, whose ultraviolet and fluorescence characteristics were basically consistent with those of Multivitamin Iron Oral Solution. Ultra-performance liquid chromatography-quadrupole-time-of-flight tandem mass spectrometry (UPLC-Q-TOF-MS/MS) analysis confirmed that P1 contained characteristic fragments of conjugated unsaturated structure, which was the key chromophore responsible for its fluorescence properties. In summary, this study explored the main browning mechanism of Multivitamin Iron Oral Solution. It was found that after citric acid catalyzed the hydrolysis of sucrose, the generated reducing sugars underwent Maillard reaction with lysine to produce fluorescent color-forming substances, and heat treatment significantly aggravated the browning process. The results of this study not only provide a solid theoretical basis for optimizing the preparation process and improving the storage stability of Multivitamin Iron Oral Solution, but also offer an important reference for the research on the browning mechanism and stability of other sugar-containing liquid preparations.

## 1. Introduction

Multivitamin Iron Oral Solution is a commonly used oral nutritional supplement in clinical practice, which is widely used for the prevention and treatment of iron deficiency anemia. However, during storage, it often exhibits a phenomenon of gradual color darkening, changing from reddish-brown to blackish-brown. This not only fails to meet the description of “reddish-brown” for properties in quality standards, but also may indicate the deterioration of product quality and the generation of potential harmful substances [1]. In addition, in previous drug sampling inspections, we found that the average content of 5-hydroxymethylfurfural in Multivitamin Iron Oral Solution was significantly higher than that in other sugar-containing preparations, which also prompted us to conduct in-depth research on the correlation between its browning mechanism and 5-HMF.

5-HMF is a common product of the Maillard reaction, which is considered a potential harmful substance in food and drugs and may pose a threat to human health [2,3]. The Maillard reaction is a typical non-enzymatic browning reaction, which mainly occurs between the carbonyl groups of reducing sugars and the amino groups of amino compounds such as amino acids and proteins. During the reaction process, a series of colored and colorless Maillard reaction products (MRPs) are generated, including small-molecule substances such as furans, pyrazines, and pyrroles, as well as macromolecular polymers such as melanoidins [4]. These products have important effects on the color, flavor, nutritional value, and safety of food and drugs, and the generation process of MRPs is also affected by factors such as temperature, time, pH value, and component ratio [5,6,7,8].

Importantly, browning in liquid pharmaceutical formulations, such as oral solutions, is not merely a sensory esthetic issue but a critical stability concern. It can lead to changes in chemical composition, the formation of toxicologically relevant impurities, and consequently, may compromise the therapeutic efficacy and patient compliance of the drug product [9,10,11]. Therefore, understanding the underlying mechanism of color deterioration is essential for ensuring the quality and safety of liquid dosage forms.

The main components of Multivitamin Iron Oral Solution include a large amount of sucrose and lysine, as well as excipients such as citric acid, niacin, and folic acid, which are potential substrates or catalysts for the Maillard reaction and sucrose hydrolysis [12,13,14]. As the main sugar component, sucrose is prone to hydrolysis under acidic or heating conditions to generate glucose and fructose, which can further undergo the Maillard reaction with amino acids [15]. Previous studies have confirmed that the Maillard reaction between reducing sugars such as glucose, fructose, ribose, and amino acids [16,17] is an important cause of browning in sugar-containing foods and preparations, and 5-HMF, as a key intermediate product of this reaction, has a good correlation with the degree of browning [18]. In the context of liquid pharmaceutical preparations, drug-excipient interactions involving Maillard reactions have been documented as a significant stability risk, particularly in formulations containing reducing sugars or sucrose that can hydrolyze to generate reactive carbonyl sources [19]. However, the specific browning mechanism of Multivitamin Iron Oral Solution, the key components involved in the reaction, and the structural characteristics of colored Maillard reaction products remain unclear. In particular, the synergistic effect of the acidic excipient citric acid on sucrose hydrolysis and subsequent Maillard reaction has not been systematically studied.

Therefore, in-depth exploration of the browning mechanism of Multivitamin Iron Oral Solution, and clarification of the substances causing color darkening and their formation pathways, are of great significance for improving product quality standards and optimizing production processes. Through the detection of commercially available oral solutions and the construction of simulated model systems, this study systematically investigated the synergistic effect of citric acid, sucrose, and lysine in the browning process, and separated and structurally characterized the Maillard products. This study not only addresses a specific stability concern in a liquid formulation context, but also clarified the browning mechanism of Multivitamin Iron Oral Solution, and provided a theoretical basis for improving the storage stability of this preparation, optimizing the formulation process and formulating more stringent quality control standards, and also offered an important reference for the stability research of other sugar-containing liquid preparations.

## 2. Results

### 2.1. Changes in Related Parameters of Multivitamin Iron Oral Solution with Storage Time

The contents of 5-HMF, sucrose, glucose, and fructose were determined using validated HPLC methods. For 5-HMF, good linearity was obtained in the range of 2.4600–98.4000 μg·mL^−1^ (r = 0.9999), with a limit of detection (LOD) of 0.37 μg·mL^−1^ and a limit of quantification (LOQ) of 1.23 μg·mL^−1^. The instrumental precision RSD was 0.08%, repeatability RSD was 0.44%, and solution stability RSD within 24 h was 0.06%. For saccharides, good linearity was achieved in the range of 3.1020–827.2000 μg·mL^−1^ (r = 0.9999) for sucrose, 52.9470–847.1520 μg·mL^−1^ (r = 0.9999) for glucose, and 52.6473–842.3568 μg·mL^−1^ (r = 0.9999) for fructose. The LOD and LOQ were 13.51 μg·mL^−1^ and 45.03 μg·mL^−1^ for sucrose, 24.36 μg·mL^−1^ and 81.21 μg·mL^−1^ for glucose, and 24.26 μg·mL^−1^ and 80.87 μg·mL^−1^ for fructose, respectively. The instrumental precision RSDs were 0.69%, 0.53%, and 0.75%, repeatability RSDs were 0.89%, 0.14%, and 1.07%, and solution stability RSDs within 24 h were 0.27%, 0.41%, and 0.35%, respectively. All validation results confirmed that the analytical methods were accurate, precise, stable, and reliable.

**Figure 1:**
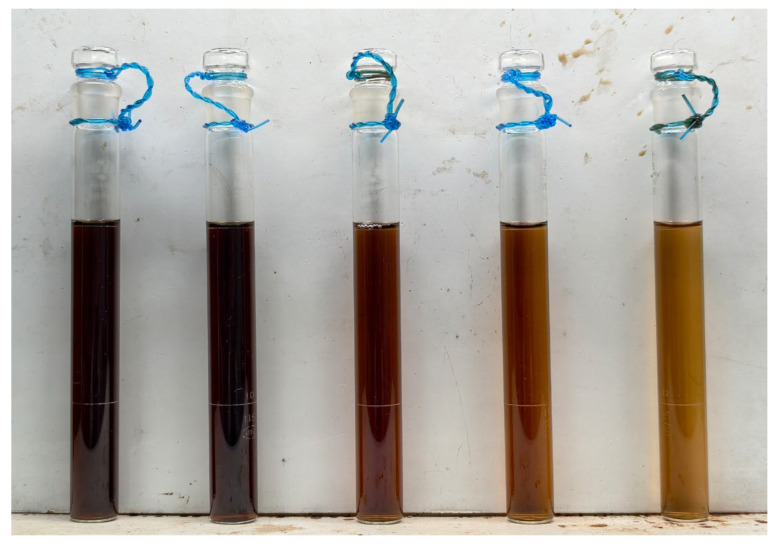
Color changes in Multivitamin Iron Oral Solution samples with storage time. From left to right: samples stored at room temperature for 24, 18, 12, 10, and 6 months, respectively.

By comparing the color changes in Multivitamin Iron Oral Solution samples from different batches, the preparation was found to gradually darken in color and even turn blackish-brown with prolonged storage time (Figure 1).

The content determination results showed that the 5-HMF content in Multivitamin Iron Oral Solution increased significantly with prolonged storage time (*p* < 0.05), which was basically consistent with the continuous increase in 5-HMF content with storage time reported in the literature [20]. The sucrose content in Multivitamin Iron Oral Solution showed a downward trend with prolonged storage time, while the contents of glucose and fructose increased significantly (*p* < 0.05) (Table 1).

**Figure 2:**
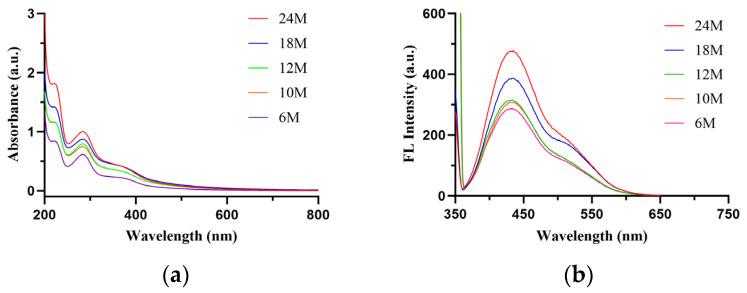
Overlaid UV-fluorescence emission spectra of Multivitamin Iron Oral Solution. (**a**) UV spectrum; (**b**) Fluorescence spectrum.

Ultraviolet spectral scanning revealed that the oral solution exhibited obvious absorption peaks in the ultraviolet region (200–400 nm), with a maximum absorption peak near 280 nm, and the absorbance increased with prolonged storage time; the absorption in the visible region (400–800 nm) was relatively weak (Figure 2a). Fluorescence spectral scanning showed that the oral solution had a maximum fluorescence emission peak near 430 nm, and the fluorescence intensity increased significantly with prolonged storage time (Figure 2b). The formation of fluorescent products is usually accompanied by browning [21], indicating that the observed color change in the oral solution may be associated with the generation of fluorescent melanoidin-like products.

### 2.2. Identification of the Color-Forming Reaction Model of Multivitamin Iron Oral Solution

#### 2.2.1. Comparison of Color Parameters Among Different Reaction Models

The four model systems were formulated according to the prescription ratio of Multivitamin Iron Oral Solution with a total volume of 50 mL, including sucrose-lysine, sucrose-citric acid, sucrose-niacin, and sucrosefolic acid model systems. Since obvious color changes were observed only after heating at 100 °C for 5 h, the browning index (BI), color density (CD), and absorbance ratio (AR) of each model under this condition were selected for comparison. Among all reaction models, the sucrose-lysine model exhibited the deepest color, with significantly higher BI and CD values and significantly lower AR values than the other models (*p* < 0.05). The sucrose-citric acid model ranked second, while no obvious color changes were observed in the sucrose-niacin and sucrose-folic acid models (Table 2). These color parameter results indicated that the sucrose-lysine reaction model was the main contributor to the increase in color intensity and browning of Multivitamin Iron Oral Solution.

#### 2.2.2. Comparison of Carbohydrate Components and 5-HMF Content in Different Reaction Models

After heating at 100 °C for 5 h, among all reaction models, the sucrose-citric acid model showed a relatively lower sucrose content, and significantly higher contents of glucose, fructose, and 5-HMF (*p* < 0.05) (Table 3). These results indicated that under the same conditions, the acidic environment (citric acid system) was more favorable for the hydrolysis of sucrose into glucose and fructose, and promoted the formation of 5-hydroxymethylfurfural (5-HMF). The hydrolysis of sucrose provided more reducing sugars as reaction substrates for the Maillard reaction, which also laid a prerequisite for the browning of the oral solution.

#### 2.2.3. Analysis of Fluorescence Emission Spectra Characteristics of Different Reaction Models

**Figure 3:**
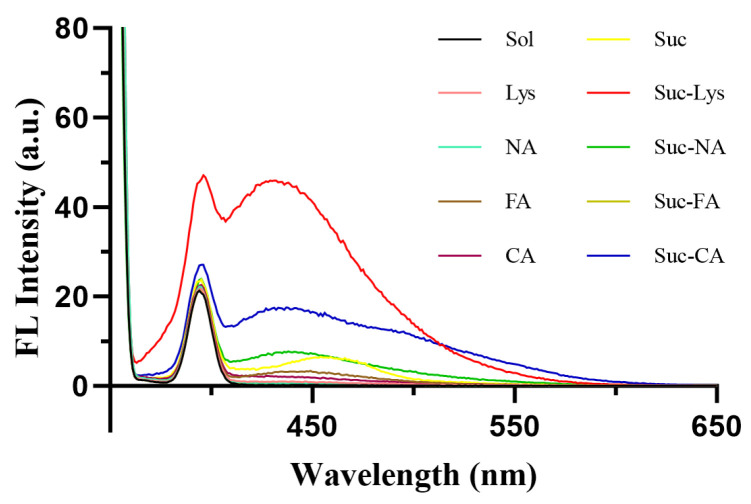
Comparison of fluorescence intensities of different reaction models. Sol: solvent (water); Lys: L-lysine hydrochloride; NA: niacin; FA: folic acid; CA: citric acid; Suc: sucrose.

Fluorescence emission spectra of the four reaction models (Figure 3) showed that the sucrose-lysine (Suc-Lys) model exhibited a strong fluorescence emission peak around 430 nm, which was basically consistent with the fluorescence characteristics of reaction products between reducing sugars and amino acids reported in the literature [22,23]. Although the other models showed slight fluorescence absorption, their maximum emission intensities were significantly weaker than that of the sucrose-lysine model, indicating that the fluorescent color-forming substances in Multivitamin Iron Oral Solution were mainly produced by the sucrose-lysine reaction. Recent studies have confirmed that the fluorescence emission peak around 430 nm is a characteristic peak of typical fluorescent intermediates in the Maillard reaction. Bin et al. [24] pointed out in their review that fluorescent compounds, as important intermediates of the Maillard reaction, can be used to construct real-time monitoring fluorescent probes, and their spectral characteristics are key indicators for analyzing the Maillard reaction process. In addition, Risum et al. [25] systematically investigated the fluorescence characteristics of Maillard reaction products in protein models using fluorescence spectroscopy combined with parallel factor analysis, and verified the reliability of fluorescent intermediates as characteristic markers of the Maillard reaction. Therefore, the 430 nm fluorescence emission peak observed in this study can be assigned to fluorescent intermediates formed during the Maillard reaction.

### 2.3. Effect of Ferric Glycerophosphate on Browning

**Figure 4:**
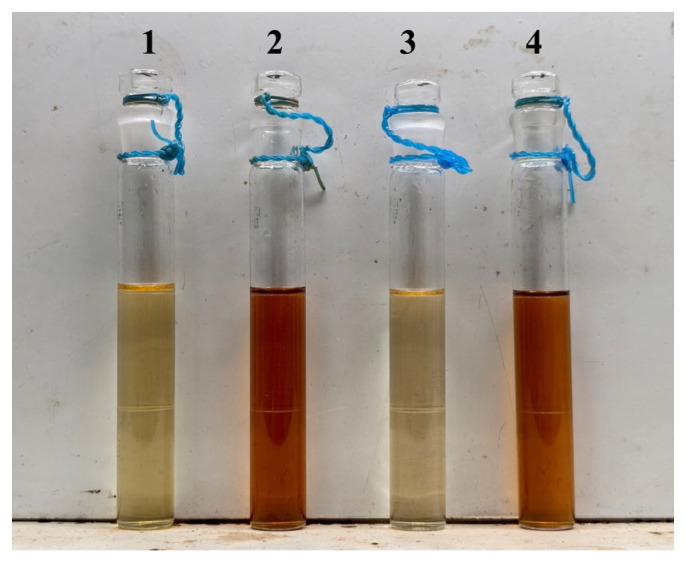
Visual color comparison of four model systems after heating. (1) Sucrose + Lysine; (2) Sucrose + Lysine + Citric Acid; (3) Sucrose + Lysine + Ferric Glycerophosphate; (4) Sucrose + Lysine + Citric Acid + Ferric Glycerophosphate.

As shown in Figure 4, Groups 1 and 3 (without citric acid) exhibited only slight yellowing, while Groups 2 and 4 (with citric acid) showed significant and similar browning. This result confirmed that ferric glycerophosphate had a negligible catalytic effect on the browning reaction, and citric acid was the dominant factor driving color deepening.

### 2.4. Investigation of Influencing Factors and Parameter Correlation Analysis of the Color-Forming Reaction Model

**Figure 5:**
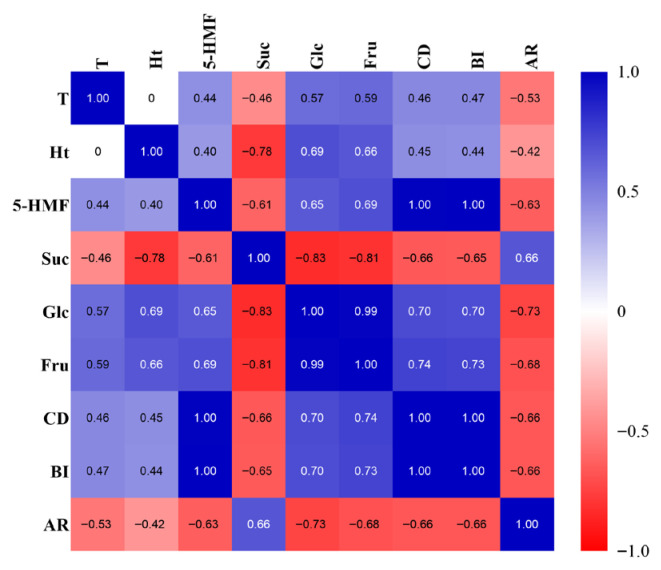
Correlation heatmap of various reaction conditions and parameters. Correlation analysis was based on 8 independent experimental groups (n = 3 per group). T: heating temperature; Ht: heating time; 5-HMF: 5-hydroxymethylfurfural content; Suc: sucrose content; Glc: glucose content; Fru: fructose content; BI: browning index; CD: color density; AR: absorbance ratio. Blue indicates positive correlation, red indicates negative correlation, and color depth represents the strength of correlation.

Based on the above results, the sucrose-lysine reaction model was identified as the main color-forming reaction model of Multivitamin Iron Oral Solution. Correlation analysis was performed using the mean values of eight independent experimental groups, with three parallel replicates for each group. The correlations of heating time and heating temperature with BI, CD, AR, and the contents of sucrose, glucose, fructose, and 5-HMF in this model under corresponding conditions were investigated. As shown in the correlation heatmap (Figure 5), heating temperature (T) and heating time (Ht) were significantly positively correlated with 5-HMF, glucose (Glc), fructose (Fru), and browning indices (CD, BI), and significantly negatively correlated with sucrose (Suc) and AR. 5-HMF was highly positively correlated with CD and BI (r = 0.996, 0.997, *p* < 0.05), significantly positively correlated with Glc and Fru, and significantly negatively correlated with Suc and AR. Glc was extremely strongly positively correlated with Fru (r = 0.988, *p* < 0.05). AR was significantly negatively correlated with 5-HMF, CD, BI, Glc, and Fru, and significantly positively correlated with sucrose. These results indicate that prolonged heating time and elevated heating temperature can promote sucrose degradation and the formation of Maillard reaction products, and color browning can serve as a visual early-warning indicator for the increase of 5-HMF content in the oral solution.

### 2.5. Results on Isolation and Characterization of Maillard Reaction Products

#### 2.5.1. Isolation Results

**Figure 6:**
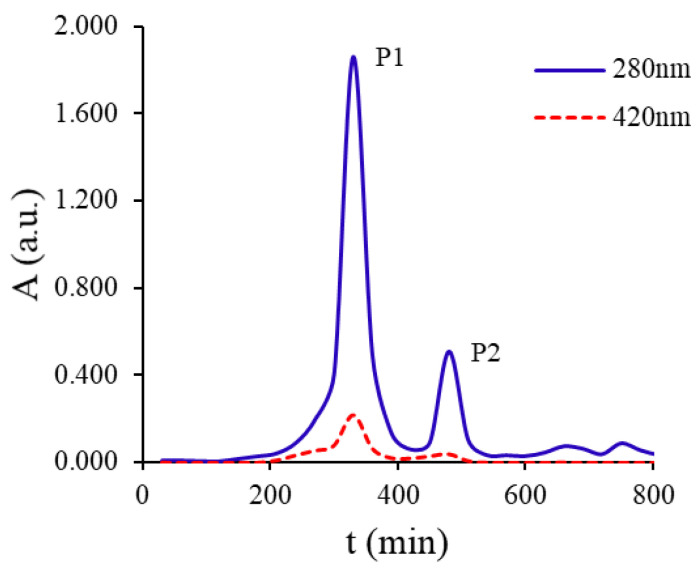
Elution curve of the collected fractions.

**Figure 7:**
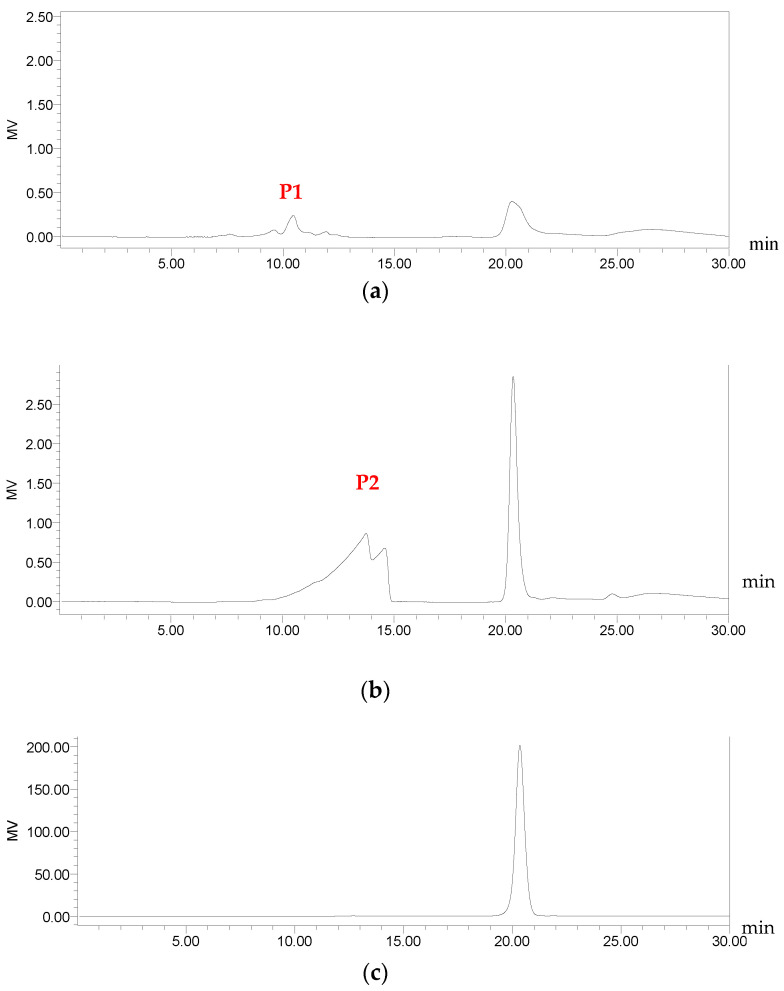
Size-exclusion chromatography profiles of fraction P1, fraction P2, and sucrose. (**a**) P1; (**b**) P2; (**c**) Sucrose.

After establishing the Maillard color-forming reaction model of sucrose-lysine, the reaction mixture was separated by Sephadex LH-20 gel column chromatography. Two main elution peaks (fractions P1 and P2, Figure 6) were detected at 280 nm, indicating that the reaction products contained at least two types of components with ultraviolet absorption. P1 eluted earlier than P2, suggesting a higher molecular weight. It also showed weak absorption at 420 nm and was presumed to be high-molecular-weight melanoidins related to browning. P2 had a lower molecular weight and only exhibited absorption at 280 nm, which may be low-molecular-weight Maillard reaction intermediates. To further confirm the molecular weight differences among sucrose, P1, and P2, size-exclusion chromatography (SEC) was performed (Figure 7).

#### 2.5.2. Molecular Weight Determination

The apparent molecular weight standard curve was established using dextran standards, with the regression equation y = −0.2511x + 7.4128, showing a good linear relationship (R^2^ = 0.9989). The isolated fractions P1 and P2 were determined, and their retention times were substituted into the curve equation. The results showed that the apparent molecular weight of fraction P1 was 61,660 Da, and that of fraction P2 was 9120 Da. These values are apparent molecular weights estimated based on dextran calibration, rather than absolute average molecular weights (Mw), due to the heterogeneous molecular weight distribution of melanoidins.

#### 2.5.3. UV and Fluorescence Spectral Characterization

**Figure 8:**
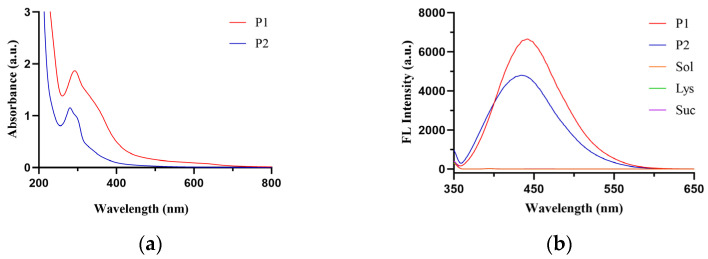
UV-fluorescence emission spectra of fractions P1 and P2. (**a**) UV spectrum; (**b**) Fluorescence spectrum.

**Figure 9:**
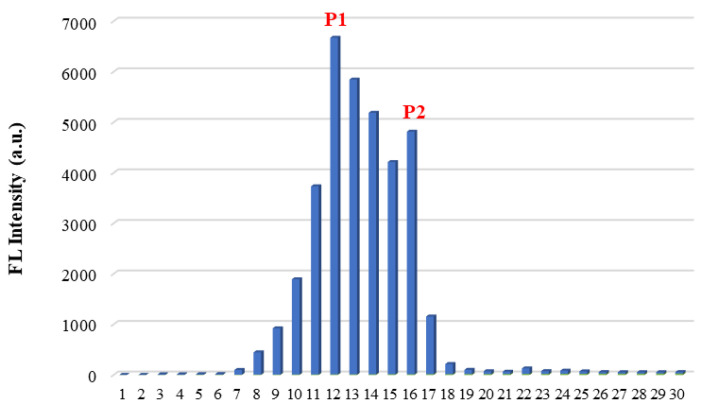
Fluorescence intensities of all collected fractions.

UV spectral scanning revealed that both fractions P1 and P2 had a maximum absorption peak near 280 nm, with the absorbance of P1 being higher than that of P2 (Figure 8). Fluorescence spectral scanning showed that both fractions P1 and P2 exhibited fluorescence absorption at 430 nm. Among all collected fractions, fraction P1 showed relatively strong fluorescence absorption, indicating a relatively high concentration of fluorescent color-forming substances (Figure 9).

#### 2.5.4. Structural Elucidation by UPLC-Q-TOF-MS/MS

Based on the above experimental results, fraction P1, with its relatively high molecular weight and the strongest UV absorption and fluorescence characteristics, was considered the most likely component corresponding to melanoidins, the typical products of the Maillard reaction. To clarify the molecular basis of color-forming substances in the sucrose-lysine model, P1 was selected for structural elucidation using UPLC-Q-TOF-MS/MS.

Primary MS analysis showed that the main characteristic peaks of fraction P1 appeared at *m*/*z* 827, 425, and 329. Among them, *m*/*z* 425 exhibited the highest signal intensity and displayed a complete and reasonable MS/MS fragmentation pattern, and was thus identified as the protonated quasi-molecular ion ([M + H]^+^). *m*/*z* 329 was attributed to a typical dehydrated intermediate formed during the Amadori rearrangement and degradation of sucrose-lysine conjugates, while *m*/*z* 827 was tentatively assigned as a dimeric or aggregated species ([2M + H]^+^) derived from low-molecular-weight Maillard products.

**Figure 10:**
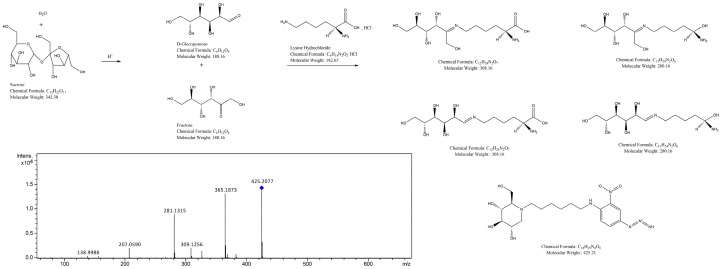
MS/MS spectrum of *m*/*z* 425.

Collision-induced dissociation of *m*/*z* 425 in the MS/MS mode generated characteristic fragment ions at *m*/*z* 365, 309, 281, 207, and 138; the corresponding MS/MS spectrum is shown in Figure 10. Combined with the Maillard reaction mechanism, sucrose was first hydrolyzed into glucose and fructose in the reaction system, followed by amino-carbonyl condensation with lysine. In the MS/MS spectrum, the parent ion *m*/*z* 425 first lost a neutral fragment of 60 Da to form *m*/*z* 365, and then further lost 56 Da to produce *m*/*z* 309, which corresponded to the typical early-stage product formed by the addition and Amadori rearrangement of glucose, fructose, and lysine. Subsequently, *m*/*z* 309 lost 28 Da through decarbonylation to generate the key intermediate *m*/*z* 281. Further cleavage of the glycosyl side chain and skeleton fragmentation of this intermediate could produce stable small-molecule fragments such as *m*/*z* 207 and 138, most of which contained conjugated unsaturated double bonds and nitrogen-containing functional groups, presumed to be important structural units contributing to the fluorescence properties in fraction P1. Based on the UV-fluorescence response and mass spectral characteristics, fraction P1 could be preliminarily identified as the key component dominating color formation in the sucrose-lysine model.

## 3. Discussion

Multivitamin Iron Oral Solution is widely used in clinical practice and shows favorable iron-supplementing efficacy and good patient tolerance. However, the phenomenon of color deepening and the gradual change from reddish-brown to blackish-brown during storage at room temperature has become an important practical issue that affects the appearance quality of the pharmaceutical product and patient medication compliance [26]. Such browning is not only related to the sensory evaluation of the preparation, but also may indicate sugar degradation, impurity formation, and potential quality risks [27]. In the present study, the browning degree of the oral solution was visually evaluated and recorded during storage, accompanied by quantitative determination of sucrose, glucose, fructose, and 5-HMF contents. These indicators reflected the degradation of sugar components and the formation of Maillard-related impurities, thus objectively revealing the quality changes in the preparation during storage. Therefore, systematic elucidation of its intrinsic chemical mechanism is of great significance for improving the stability of the preparation. It should be emphasized that the iron component in the commercial preparation is ferric glycerophosphate, a stable organic iron complex rather than free Fe^2+^ or Fe^3+^ ions. Comparative experiments using four model systems confirmed that ferric glycerophosphate exerted a negligible catalytic effect on the browning reaction, whereas color deepening was only observed in systems containing citric acid. Therefore, a simulated reaction system consisting of three components (citric acid, sucrose, and lysine) was established in this study. Combined with kinetic monitoring, spectral characterization, and structural analysis by high-resolution mass spectrometry, the chemical nature of browning was clarified: citric acid catalyzes the hydrolysis of sucrose to release reducing sugars, which then undergo the Maillard reaction with lysine, resulting in the formation of fluorescent color-producing products.

Citric acid, as an acidic excipient in Multivitamin Iron Oral Solution, exhibits a catalytic effect during the browning process, which is one of the key findings of this study. The results showed that under the same condition (100 °C for 5 h), sucrose was almost completely hydrolyzed in the sucrose-citric acid model, and the contents of glucose, fructose, and 5-HMF were significantly higher than those in other models, confirming that an acidic environment can remarkably promote the acid-catalyzed hydrolysis of sucrose. As a non-reducing disaccharide, sucrose has its anomeric carbon locked by an α-1,β-2 glycosidic bond and cannot directly participate in the Maillard reaction [28]. By lowering the pH of the system and markedly increasing the proton concentration, citric acid induces acid-catalyzed hydrolysis of the glycosidic bond, thereby releasing glucose and fructose with free aldehyde or ketone groups, which provide the necessary reducing sugar substrates for the subsequent Maillard reaction. This mechanism is generally consistent with reports in the literature that acidic environments accelerate carbohydrate hydrolysis [29], and also explains why the 5-HMF content in Multivitamin Iron Oral Solution is significantly higher than that in other sugar-containing liquid preparations without citric acid. Citric acid improves the efficiency of sucrose hydrolysis, which not only promotes the occurrence of the Maillard reaction but also facilitates the substantial formation of 5-HMF, an intermediate product of the caramelization reaction. Furthermore, 5-HMF is highly positively correlated with the browning index and color density, confirming that it can be used as an intuitive evaluation index for the browning degree of this preparation.

With a sufficient supply of reducing sugars, lysine serves as the key nitrogen source that determines the browning direction and color development characteristics. In this study, by comparing four simulated reaction models, the sucrose-lysine reaction was identified as the main reaction pathway responsible for the browning of Multivitamin Iron Oral Solution. Compared with the reaction models of sucrose with citric acid, niacin, folic acid, and the blank group, the sucrose-lysine model exhibited significantly higher browning index and color density, and its fluorescence intensity peaked at 430 nm, which was highly consistent with the fluorescence characteristics of the commercial preparation. This confirms that the fluorescent color-forming substances in the preparation are mainly produced by the Maillard reaction between sucrose hydrolysates and lysine. In contrast, no obvious browning or strong fluorescence signal was observed in the niacin and folic acid models, indicating that other nitrogen-containing excipients in the preparation are not key components involved in browning, further highlighting the dominant role of lysine in the Maillard reaction. The lysine molecule contains two basic amino groups (α-amino and ε-amino), which retain high nucleophilic activity even in weakly acidic environments. It can rapidly condense with the open-chain carbonyl groups of glucose or fructose to form Schiff bases, which then undergo Amadori rearrangement to generate stable adducts, thereby initiating the Maillard reaction [30,31]. The early products generated via this pathway are rich in conjugated enones, imines, and five-membered heterocyclic structures, which form the structural basis of fluorescent chromophores—this is the main reason why its fluorescence intensity is significantly higher than that of other models [32]. Studies have shown that fluorescent compounds, as browning precursors, accumulate in the early stage of the Maillard reaction and can be further degraded into dark-colored substances [33]; the results of the present study are generally consistent with those reports.

Heating temperature and time are key external factors that accelerate the browning process. Correlation analysis showed that heating time and temperature were significantly positively correlated with reducing sugar content, 5-HMF content, and browning indices, and significantly negatively correlated with sucrose content (*p* < 0.05). This indicates that heating plays dual roles in accelerating browning: on the one hand, it promotes citric acid-mediated sucrose hydrolysis and increases the production of reducing sugars [34]; on the other hand, it provides activation energy for each stage of the Maillard reaction, significantly accelerating reaction rates such as amino-carbonyl condensation, Amadori rearrangement, dehydration, and cyclization. The negative correlation between absorbance ratio and browning indices suggests that with the progression of the reaction, the color-forming substances in the preparation are dominated by small-molecular fluorescent intermediates, which also points out the direction for the subsequent isolation and characterization of browning products. In addition, the high correlation between color browning and 5-HMF content indicates that preliminary early warning of the storage stability of Multivitamin Iron Oral Solution can be achieved by rapidly detecting 5-HMF content or directly observing color changes.

To further clarify the molecular basis of fluorescent coloration in the preparation, Sephadex LH-20 gel column chromatography was employed to separate the browning solution, yielding the major fluorescent fraction P1. This fraction exhibited strong UV absorption at 280 nm and 420 nm, with a maximum fluorescence emission at approximately 430 nm upon excitation at 347 nm, which was generally consistent with the spectral characteristics of the preparation. Its apparent molecular weight was determined to be 61,660 Da via Sephadex LH-20; this value is derived from hydrodynamic volume and adsorption behavior, representing the apparent characteristic of Maillard reaction products in the form of aggregates, and is thus categorized as a high-molecular-weight Maillard reaction product fraction.

UPLC-Q-TOF-MS/MS analysis revealed *m*/*z* 425 as the major quasi-molecular ion peak in the primary mass spectrum of fraction P1, with characteristic fragment ions including *m*/*z* 309, 281, 207, and 138 generated via further fragmentation in the MS/MS spectrum. This analysis was performed under dilute and polar mobile phase conditions, where non-covalent aggregates dissociate into individual structural units; thus, the aforementioned *m*/*z* signals represent the core molecular structure of the individual structural units in P1. Among these ions, *m*/*z* 309 is a typical early-stage Maillard reaction product formed by the Amadori rearrangement of glucose/fructose with lysine, and *m*/*z* 281 is a key intermediate derived from the dehydration and decarbonylation of this product. Small-molecule fragment ions such as *m*/*z* 207 and *m*/*z* 138 are rich in α,β-unsaturated carbonyl and nitrogen-containing heterocyclic structures, and these conjugated systems are the key chromophores responsible for the strong fluorescence properties of P1 [35,36]. In summary, P1 is not a covalently linked high-molecular-weight polymer of approximately 61 kDa, but a non-covalent aggregate formed by low- and medium-molecular-weight Maillard reaction products through hydrophobic interactions, hydrogen bonding, or π-π stacking. This conclusion is consistent with the results of both gel chromatography and mass spectrometry analyses, and clarifies the chemical nature of fraction P1.

The above results also indicated that the browning of Multivitamin Iron Oral Solution was not conventional coloration in the visible region, but was dominated by fluorescence-based coloration. The medium and small molecular weight nitrogen-containing conjugated compounds produced by the Maillard reaction undergo electronic transition and emit visible light under excitation at a specific wavelength; the superposition and accumulation of such emission lead to the visually discernible deepening of brownish discoloration [37,38]. This finding revises the traditional cognitive framework that only focuses on macroscopic color changes, reveals the nature of coloration at the molecular fluorescence mechanism level, and provides a new perspective for the stability study of sugar-containing liquid preparations.

Several aspects of this study deserve further investigation. The present work only explored the roles of citric acid, sucrose, and lysine in browning. Whether ferric ions in the preparation undergo complexation or redox reactions with Maillard reaction intermediates such as catechol-like compounds and α-dicarbonyl compounds [39], thereby affecting the browning rate and product distribution, has not been thoroughly examined.

In addition, structural characterization of fraction P1 was limited to fragment analysis, and the complete molecular structure has not been identified.

Future studies may further explore the interaction between ferric ions and Maillard reaction products to clarify the mechanism underlying the browning of the preparation. Highly pure fraction P1 can be obtained via preparative liquid chromatography, and its complete structure can be identified by combining ^1^H NMR and ^13^C NMR spectroscopy [40], to provide a more precise theoretical basis for the development of anti-browning preparations.

## 4. Experimental Methods

### 4.1. Materials and Instruments

Multivitamin Iron Oral Solution (commercially available, purchased from different manufacturers); sucrose (purchased from Merck Millipore, Darmstadt, Germany), L-lysine hydrochloride, niacin, folic acid (purchased from Shanghai Macklin Biochemical Technology Co., Ltd., Shanghai, China); citric acid (purchased from Guangdong Guanghua Sci-Tech Co., Ltd., Shantou, China); Ferric glycerophosphate (purchased from Sichuan Bastie Fine Chemical Co., Ltd., Meishan, China); 5-hydroxymethylfurfural, glucose, fructose and dextran standards (purchased from the National Institute for Food and Drug Control, Beijing, China); sodium 1-heptanesulfonate (purchased from Shandong Keyuan Biochemical Co., Ltd., Heze, China); phosphoric acid and triethylamine (purchased from Taicang Hushi Reagent Co., Ltd., Taicang, China); acetic acid (purchased from Tianjin Kemiou Chemical Reagent Co., Ltd., Tianjin, China); Sephadex LH-20 (purchased from Pharmacia Biotech AB, Uppsala, Sweden); acetonitrile (purchased from Guangdong Xilong Scientific Co., Ltd., Shantou, China). Acetonitrile was of chromatographic grade, and all other reagents were of analytical grade. Water used in the experiments was ultrapure water.

High-performance liquid chromatography (HPLC) (Waters Corporation, Milford, MA, USA); UV-Vis spectrophotometer and fluorescence spectrophotometer (Shimadzu Corporation, Kyoto, Japan); ultra-performance liquid chromatography-quadrupole-time-of-flight tandem mass spectrometry (UPLC-Q-TOF-MS/MS) (Bruker Corporation, Billerica, MA, USA); constant-temperature water bath (Julabo Technology (Beijing) Co., Ltd., Beijing, China); forced-air drying oven (Binder GmbH, Tuttlingen, Germany); pH meter and analytical balance (Mettler-Toledo, Greifensee, Switzerland).

### 4.2. Determination of 5-HMF Content

5-HMF in Multivitamin Iron Oral Solution samples with different storage times was determined by high-performance liquid chromatography with ultraviolet detection (HPLC-UV). An Xltimate C_18_ column (250 mm × 4.6 mm, 5 μm; Welch Materials (Shanghai) Co., Ltd., Shanghai, China) was used. Mobile phase A was 0.005 mol·L^−1^ sodium 1-heptanesulfonate solution containing 0.025% triethylamine, adjusted to pH 4.5 with 20% glacial acetic acid, and mobile phase B was acetonitrile. The flow rate was 1.0 mL·min^−1^, the detection wavelength was 284 nm, the column temperature was set at 40 °C, and the injection volume was 20 μL. The determination was performed according to the following elution program: 0–10 min: 97% A; 10–45 min: 97% A; 45–48 min: 48% A; 48–55 min: 97% A.

### 4.3. Determination of Carbohydrate Contents

The contents of sucrose, glucose, and fructose in the solution were determined by high-performance liquid chromatography with refractive index detection (HPLC-RID). Exactly 10 mg each of sucrose, fructose, and glucose reference substances were transferred into 10 mL volumetric flasks (10-fold dilution), and 0.1 mL of Multivitamin Iron Oral Solution sample was placed into a 100 mL volumetric flask (1000-fold dilution). A SHODEX SUGAR SC1011 column (300 mm × 8.0 mm, 6 μm; Shodex, Tokyo, Japan) was used with water as the mobile phase. The flow rate was 0.35 mL·min^−1^, the column temperature was 65 °C, a refractive index detector was used with a detector temperature of 40 °C, and the injection volume was 10 μL.

### 4.4. Determination of Color Evaluation Parameters

#### 4.4.1. Color Dilution Factor

The colored original reaction system was diluted 1:1 with ultrapure water. Precisely 1.5 mL of the original reaction solution was transferred into an EP tube, mixed with 1.5 mL of ultrapure water, and the dilution count was recorded as 1. Then, 1.5 mL of the mixed solution was transferred into a new EP tube and compared with an EP tube containing 1.5 mL of ultrapure water. If the color was still darker, further dilution was performed until no color difference was observed. The total number of dilutions was defined as the color dilution factor [41].

#### 4.4.2. Browning Index, Color Density, and Absorbance Ratio

Each original reaction mixture was diluted to an appropriate concentration so that the absorbance values at 420 nm and 520 nm were within the linear detection range of the instrument. A UV-Vis spectrophotometer was used to measure absorbance at 420 nm and 520 nm. Color Density (CD), Browning Index (BI), and Absorbance Ratio (AR) were calculated using the following equations:

BI = A_420_ × DF, (1)

CD = (A_420_ + A_520_) × DF, (2)

AR = A_520_/A_420_, (3)

where A_420_ is the absorbance at 420 nm, A_520_ is the absorbance at 520 nm, and DF is the color dilution factor. The CD value represents the color intensity of the solution; a higher CD indicates a darker color. The BI value reflects the browning degree of the solution; a higher BI indicates more severe browning. The AR value reflects the structural characteristics of color-producing substances. A higher AR value indicates a larger proportion of absorption at 520 nm, suggesting more complex conjugated double-bond systems and higher molecular weights (e.g., melanoidins). A lower AR value indicates a larger proportion of absorption at 420 nm, meaning the color-producing substances are dominated by small-molecule browning intermediates [42].

### 4.5. Spectral Analysis

The solution was diluted to an appropriate concentration, and UV-Vis full-wavelength scanning (200–800 nm) was performed using a UV-Vis spectrophotometer. The position of the maximum absorption peak (λmax) and the corresponding absorbance value were recorded.

Fluorescence emission spectra were recorded using a fluorescence spectrophotometer with an excitation wavelength of 347 nm. The scanning wavelength range was 350–650 nm, with an excitation slit of 5 nm, an emission slit of 10 nm, a scanning rate of 1200 nm/s, and a photomultiplier tube voltage of 500 V. The emission spectrum and maximum fluorescence intensity were recorded.

### 4.6. Determination of the Color Reaction Model of Multivitamin Iron Oral Solution

According to the formulation of Multivitamin Iron Oral Solution (50 mL volume), four reaction model solutions were prepared separately: sucrose-lysine, sucrose-citric acid, sucrose-niacin, and sucrose-folic acid models. The main color reaction model was screened by comparing color parameters, carbohydrate contents, 5-HMF content, and spectral characteristics after heating at 100 °C for 5 h. Meanwhile, to clarify the catalytic effect of ferric glycerophosphate on the browning process, four additional comparative model systems were designed: (1) sucrose + lysine, (2) sucrose + lysine + citric acid, (3) sucrose + lysine + ferric glycerophosphate, and (4) sucrose + lysine + citric acid + ferric glycerophosphate. All these model solutions were treated under the same heating condition (100 °C for 5 h), and their color changes were observed and recorded.

### 4.7. Investigation of Influencing Factors and Correlation Analysis of Parameters in the Color Reaction Model of Multivitamin Iron Oral Solution

The color reaction model of Multivitamin Iron Oral Solution was prepared according to its formulation (50 mL volume). The effects of different heating conditions on 5-HMF, sucrose, glucose, fructose, BI, CD, and AR were investigated, and correlation analysis among these parameters and conditions was performed.

Effect of heating temperature: The initial pH of the solution was adjusted to 7 with 0.2 mol·L^−1^ HCl or 0.2 mol·L^−1^ NaOH. Samples were taken after water bath heating at 70 °C, 80 °C, 90 °C, and 100 °C for 5 h.

Effect of heating time: The initial pH of the solution was adjusted to 7 with 0.2 mol·L^−1^ HCl or 0.2 mol·L^−1^ NaOH. Samples were taken after water bath heating at 100 °C for 0.5 h, 1 h, 3 h, and 5 h.

### 4.8. Preparation of Maillard Reaction Products (MRPs)

Appropriate amounts of sucrose and L-lysine hydrochloride were weighed and mixed at a molar ratio of 1:1. The mixture was dissolved in ultrapure water, and the pH was adjusted to 10 with 2 mol·L^−1^ NaOH solution (studies have shown that alkaline conditions are more favorable for the directional preparation of high-yield MRPs for separation and characterization [43]). The solution was gently heated to boiling and kept at a slight boil until the water had evaporated. The mixture was then placed in a preheated forced-air drying oven at 125 °C for 2 h, removed, and quickly cooled to room temperature in an ice-water bath. The resulting brown liquid was the MRPs [44,45]. It was sealed and stored in a refrigerator at 4 °C for further use.

### 4.9. Isolation and Characterization of Maillard Reaction Products

#### 4.9.1. Preliminary Isolation of MRPs

Sephadex LH-20 gel column chromatography was used to isolate the prepared MRPs. Exactly 2 g of sucrose-lysine Maillard reaction products was weighed into a 10 mL volumetric flask, dissolved in ultrapure water to the mark, and mixed thoroughly. The solution was filtered through a 0.45 μm aqueous filter membrane, and 3 mL of the filtrate was slowly loaded onto the gel column for separation. Ultrapure water was used as the eluent. The eluate was collected in graduated stoppered test tubes (4 mL per tube). The absorbance of the eluate at 280 nm and 420 nm was measured by UV-Vis spectrophotometer. Fractions with the highest absorbance (designated as Fraction P1 and P2) were selected for subsequent characterization.

#### 4.9.2. Determination of Molecular Weight

Gel permeation chromatography (GPC) was employed to determine the molecular weight. A series of dextran standards was analyzed to establish a standard curve of retention time versus molecular weight; the molecular weight of the isolated fractions was calculated based on their retention times [46]. Instrument parameters: Waters 2489 (Waters Corporation, Milford, MA, USA); column: TSKgel G2000SWXL (300 mm×7.8 mm, 5 μm Tosoh Corporation, Tokyo, Japan); detector: refractive index detector; detector temperature: 30 °C; column temperature: 30 °C; mobile phase: 100% ultrapure water; flow rate: 0.6 mL·min^−1^; injection volume: 20 μL.

#### 4.9.3. Spectral Analysis of Fractions

UV-Vis full-wavelength scanning and fluorescence spectroscopy were performed on the collected fractions to analyze their UV absorption and fluorescence emission characteristics.

#### 4.9.4. Structural Elucidation

UPLC-Q-TOF-MS/MS was used for qualitative analysis of Fraction P1, and the structural characteristics of its main fragments were identified by database searching. Chromatographic conditions: Agilent SB-C_18_ column (4.6 mm × 100 mm, 2.7 μm; Agilent Technologies, Santa Clara, CA, USA); mobile phase: 0.1% formic acid aqueous solution (A)-acetonitrile (B); gradient elution: 0–3 min, 5% B; 3–18 min, 5–95% B; 18–22.5 min, 95% B; 22.5–23 min, 95–5% B; 23–30 min, 5% B. Flow rate: 0.6 mL·min^−1^; column temperature: 35 °C; injection volume: 5 μL. Mass spectrometry conditions: electrospray ionization (ESI) source in positive and negative ion modes; capillary voltage: 2500 V; nebulizing gas pressure: 2 bar; drying gas flow rate: 8.0 L/min; drying gas temperature: 200 °C; full scan and auto MS/MS modes; mass range: *m*/*z* 50–2500. Before sample analysis, the mass axis was calibrated with sodium formate tuning solution to ensure mass accuracy error below 5 ppm.

### 4.10. Statistical Analysis

All experiments were performed in triplicate. Statistical analysis was carried out using R 4.5.1, and experimental data were expressed as “mean ± standard deviation”. *p* < 0.05 was considered statistically significant. Graphs were plotted using R 4.5.1.

## 5. Conclusions

In summary, this study clarified that the browning mechanism of Multivitamin Iron Oral Solution is mainly as follows: citric acid promotes the hydrolysis of sucrose into reducing sugars, which then undergo the Maillard reaction with lysine to produce fluorescent color-developing substances dominated by conjugated unsaturated structures. Moreover, heating can significantly accelerate this process. This finding reveals the primary cause of browning in the preparation, provides a clear direction for the process optimization of Multivitamin Iron Oral Solution, and also offers a reference for the quality research of other related sugar-containing liquid preparations.

Based on the browning mechanism elucidated above, the formulation and storage conditions of the preparation can be optimized from the following two aspects to inhibit browning. In terms of formulation design, it is recommended to optimize the amount of citric acid and reduce its concentration to an appropriate level while ensuring the taste and stability of the preparation, thereby alleviating sucrose hydrolysis. Meanwhile, appropriate anti-browning agents such as sulfites or amino acid compounds can be added to inhibit the formation of Maillard reaction intermediates. Metal ion chelators can also be incorporated to reduce the catalytic effect of trace metal ions. In terms of storage conditions, the heating temperature and time during production should be strictly controlled to avoid prolonged exposure to high temperatures. It is recommended that samples be stored at low temperature (2–8 °C) and protected from light to slow down the rates of sucrose hydrolysis and the Maillard reaction. In addition, light-resistant packaging materials can be used to reduce the promoting effect of light radiation on browning.

Through the above formulation optimization and appropriate control of storage conditions, sucrose hydrolysis and the Maillard reaction can be effectively inhibited, thereby delaying browning, improving the stability of Multivitamin Iron Oral Solution, and extending its shelf life.

## Figures and Tables

**Table 1:** Contents of sucrose, glucose, fructose, and 5-HMF in Multivitamin Iron Oral Solution at different storage times.

Storage Time	Sucrose (mg·mL^−1^)	Glucose (mg·mL^−1^)	Fructose (mg·mL^−1^)	5-HMF (μg·mL^−1^)
6 months	24.75 ± 1.05 ^a^	201.65 ± 4.32 ^a^	206.26 ± 2.05 ^a^	544.49 ± 7.38 ^a^
10 months	10.18 ± 1.19 ^b^	212.43 ± 5.96 ^a^	227.04 ± 7.97 ^b^	791.94 ± 9.19 ^b^
12 months	7.37 ± 0.74 ^c^	211.83 ± 8.60 ^a^	229.40 ± 2.74 ^b^	898.39 ± 11.01 ^c^
18 months	3.94 ± 0.46 ^d^	217.63 ± 2.38 ^a^	232.10 ± 1.69 ^b^	915.86 ± 19.24 ^c^
24 months	2.97 ± 0.87 ^d^	224.67 ± 8.16 ^b^	240.06 ± 3.78 ^c^	1073.75 ± 17.26 ^d^

Storage time: The period from the production date on the package of the commercial sample to the date of determination. Values are expressed as mean ± SD (n = 3). Different lowercase letters within the same column indicate significant differences at *p* < 0.05 by one-way ANOVA followed by post hoc test. All concentration data were converted back to the undiluted original commercial preparation based on the corresponding dilution factors. The samples were diluted 1000-fold for sugar determination and 25-fold for 5-HMF determination.

**Table 2:** Comparison of BI, CD, and AR values in different reaction models.

Model	BI	CD	AR
Sucrose-lysine	13.73 ± 0.92 ^a^	17.97 ± 0.84 ^a^	0.31 ± 0.03 ^a^
Sucrose-citric acid	0.40 ± 0.01 ^b^	0.61 ± 0.02 ^b^	0.54 ± 0.05 ^b^
Sucrose-niacin	0.19 ± 0.02 ^b^	0.36 ± 0.01 ^b^	0.89 ± 0.09 ^c^
Sucrose-folic acid	0.16 ± 0.02 ^b^	0.29 ± 0.01 ^b^	0.75 ± 0.10 ^c^
Sucrose (control)	0.15 ± 0.02 ^b^	0.27 ± 0.02 ^b^	0.76 ± 0.13 ^c^

Values are expressed as mean ± SD (n = 3). Different lowercase letters within the same column indicate significant differences at *p* < 0.05 by one-way ANOVA followed by post hoc test.

**Table 3:** Comparison of sucrose, glucose, fructose, and 5-HMF contents in different reaction models.

Model	Sucrose (mg·mL^−1^)	Glucose (mg·mL^−1^)	Fructose (mg·mL^−1^)	5-HMF (μg·mL^−1^)
Sucrose-lysine	297.82 ± 5.28 ^a^	58.89 ± 3.42 ^a^	56.32 ± 4.17 ^a^	31.88 ± 4.13 ^a^
Sucrose-citric acid	0.00 ± 0.00 ^b^	297.52 ± 11.08 ^b^	252.42 ± 8.71 ^b^	1504.62 ± 108.99 ^b^
Sucrose-niacin	44.28 ± 0.75 ^c^	179.94 ± 8.36 ^c^	193.85 ± 5.68 ^c^	121.14 ± 9.34 ^c^
Sucrose-folic acid	334.35 ± 7.45 ^d^	22.14 ± 2.33 ^d^	23.78 ± 2.75 ^d^	0.56 ± 0.10 ^a^
Sucrose (control)	465.62 ± 8.48 ^e^	11.03 ± 0.42 ^d^	9.05 ± 1.52 ^e^	0.00 ± 0.00 ^a^

Values are expressed as mean ± SD (n = 3). Different lowercase letters within the same column indicate significant differences at *p* < 0.05 by one-way ANOVA followed by post hoc test.

## Data Availability

The data presented in this study are available upon request from the corresponding authors.
